# Supplementary material for: Using YOLO v7 to Detect Kidney in Magnetic Resonance Imaging
Source: ArXiv. 2024 Feb 12:arXiv:2402.05817v2. Preprint. [Version 2] (PMC11188135)
Supplement: Supplement 1 [file NIHPP2402.05817v2-supplement-1.pdf]

Supplementary table 1. Scanners technical informations. In this study scans were captured using 5 different scanners with 1.5 or 3 Tesla magnetic field.

Company/Machine brand	Achieva	Aera	Avanto	Biograph_mMR	Echelon Oval	Skyra	Skyra_fit	TrioTim	Verio	Grand Total
**Hitachi**					**1**					**1**
1.5 T					1					1
**Philips Medical Systems**	**1621**									**1621**
1.5 T	1044									1044
3 T	577									577
**SIEMENS**		**3562**	**1**	**130**		**2**	**1**	**1**	**338**	**4035**
1.5 T		3562	1							3563
3 T				130		2	1	1	338	472
**Grand Total**	**1621**	**3562**	**1**	**130**	**1**	**2**	**1**	**1**	**338**	**5657**
